# Mixed Methods Analysis of State Policies on Care Planning and Family Engagement in Assisted Living

**DOI:** 10.1093/geroni/igaf122.3813

**Published:** 2025-12-31

**Authors:** Paula Carder, Erh-Chi Hsu, Lindsey Smith, Cassandra Hua, Adam Pennavaria, Eric Jutkowitz

**Affiliations:** Portland State University, Portland, Oregon, United States; Johns Hopkins University, Baltimore, Maryland, United States; Oregon Health & Science University, Portland, Oregon, United States; University of Massachusetts Lowell, Lowell, Massachusetts, United States; Oregon Health & Science University, Portland, Oregon, United States; University of Minnesota, Minneapolis, Minnesota, United States

## Abstract

Involving family in care planning is a person-centered care strategy that enhances assisted living (AL) residents, families, and staff well-being. This study analyzes AL care planning and family engagement requirements through a health services regulatory analysis of 256 AL licenses across all 50 states and DC associated with 37190 ALs in 2023. Using a mixed methods approach, we examined license requirements for the presence and qualitative context of family engagement in care planning, staff training on family involvement, and person-centered care. Nearly 80% of ALs were required to have care plans. A larger share of ALs designated for memory care (MC) had family engagement requirements (47%) compared to non-memory care ALs (32.1%). Half of licenses encourage family engagement in care planning, while the remainder used passive language about ensuring family roles or encouraging involvement in activities. Person-centered care policies were less common, appearing more often in MC licenses. Only licenses in 3 states emphasized respecting individuals’ cultural and ethnic values in care planning and staff training. Qualitative analysis identified the following categories in family engagement: providing direct care, and involvement in care planning and legal aspects (e.g., lease, payment). Person-centeredness was described in the context of care planning and staff training. The level of specificity and stringency of these categories ranged from passive to detailed and directive. These findings provide the first comprehensive profile of AL care planning requirements, establishing baseline data for future outcome evaluations.

